# Smoking Cessation Support by Text Message During Pregnancy: A Qualitative Study of Views and Experiences of the MiQuit Intervention

**DOI:** 10.1093/ntr/ntw241

**Published:** 2017-04-11

**Authors:** Melanie Sloan, Sarah Hopewell, Tim Coleman, Sue Cooper, Felix Naughton

**Affiliations:** 1 Behavioural Science Group, Institute of Public Health, University of Cambridge, Cambridge, UK;; 2 Division of Primary Care, University of Nottingham, Nottingham, UK

## Abstract

**Introduction:**

SMS text messaging is increasingly used for delivering smoking cessation support and pilot studies suggest this may also be useful in pregnancy. This study explores the views of women who received a tailored text messaging cessation intervention (MiQuit) during pregnancy, focusing on acceptability, perceived impact, and suggestions for improvements.

**Methods:**

Semi-structured interviews were undertaken with 15 purposively sampled women who had received the MiQuit intervention during pregnancy as part of a randomized controlled trial. Data were analyzed thematically.

**Results:**

Three main themes were identified: “impact”, “approach,” and “optimization.” Participants described an immediate, yet often short-lived, impact from the texts that distracted and delayed them from smoking and they perceived that texts focusing on the development of and risk to the baby generated more enduring emotional impacts. Most women found receiving support by text preferable to face-to-face cessation support, with participants citing the greater regularity, convenience, and non-judgmental style as particular advantages. Participants would have preferred a longer support program with increased tailoring, greater customization of text timings and consideration of cutting down as an alternative/precursor to quitting.

**Conclusion:**

Pregnancy-specific cessation support by text message was well received and participants considered the support increased their motivation to stop smoking. The focus on the developing baby, the regularity of contact and the provision of gentle, encouraging messages were highlighted as particularly important elements of the program.

**Implications:**

This study adds further evidence to the acceptability and perceived positive impact of text-messaging programs in aiding smoking cessation in pregnancy. The findings indicate that for some women, this type of support is preferable to face-to-face methods and could be utilized by health professionals, either in addition to current methods or as an alternative. This study is also relevant to researchers developing health-related text programs to consider participants’ desire for greater tailoring. Further research is required into adapting and continuing text support for women postpartum.

## Introduction

Smoking in pregnancy is associated with numerous health complications, including increased risks of miscarriage, pre-term labor and low birthweight.^1^ Most pregnant smokers are aware that smoking in pregnancy is harmful, although many lack detailed knowledge of associated risks.^2^ However, despite this awareness, approximately 12% of pregnant women in the United Kingdom and United States smoke throughout pregnancy.^3^^,^^4^

In the United Kingdom, pregnant smokers are offered support to quit, generally in the form of face-to-face appointments with a stop smoking service advisor. Although this has been found to be effective,^5^ and many pregnant women report wanting support to quit,^6^ take-up is low, possibly due to accessibility issues and pregnant smokers having a negative perception of these services.^7^

An alternative or complementary option is to offer pregnant smokers self-help support. Self-help materials have several advantages over face-to-face support: they are low cost, may reach women who would not attend for support in person, and are of high interest to pregnant smokers.^6^ A meta-analysis found that self-help interventions increase abstinence rates in pregnancy compared to standard care.^8^ Among non-pregnant smokers, the impact of self-help is higher among individually tailored interventions when compared to non-tailored self-help support.^9^

One increasingly common method of delivering self-help support is via SMS text messaging. Delivering cessation support by text has several benefits over many other forms of self-help as it is highly accessible, can be activated remotely, without the need for additional materials and can be synchronized to when support is likely to be most impactful. Among non-pregnant smokers, text message support has been found to be an effective intervention.^10^

Given the suitability for delivering cessation support via text to pregnant smokers, we developed a tailored text message intervention, MiQuit.^11–13^ MiQuit provides a 12-week program of text message support (mean of 0.8–1.7 per day) tailored to 13 individual characteristics.^13^ A feasibility trial found that MiQuit was acceptable to pregnant smokers, increased the likelihood of setting a quit date and increased determinants of abstinence in pregnancy, including determination to quit and self-efficacy.^11^

Although there have been several other studies on the use of self-help cessation support by text since the initial development of MiQuit, there has been limited focus on the pregnant smoker. Pregnancy is a distinct time in a woman’s life with physical, emotional and attitudinal changes that require consideration when developing a targeted cessation program. Participant views of a 5-day pilot version of MiQuit^12^ found that text support was perceived as highly convenient, which is supported by a recent investigation among pregnant women receiving a full-length text message support program.^14^ Consequently, we undertook a qualitative study to attempt to understand MiQuit recipients’ views on the text message program and how they believed this might be improved for future users.

## Methods

### Design and Procedure

MiQuit is a 12-week automated responsive tailored text message support program. Core support includes motivational messages, advice about preparing for a quit attempt, how to manage cravings, withdrawal and trigger situations, information about the development of their baby and how smoking affects babies. In addition, users can receive additional support tailored around a quit date, alter the frequency of support, and obtain instant support or distraction. See the following publication for further intervention details.^13^

This was a qualitative study using purposive sampling and the COREQ checklist for qualitative research was adhered to.^15^

### Participants

At their final follow-up (38 weeks gestation), intervention participants in the MiQuit trial were asked to consent to be contacted for an interview. Purposive sampling was carried out amongst consenting intervention participants to ensure they had a range of ages, success in quitting and parity as we thought these may influence their opinions and experience of the intervention. Participants were grouped under these criteria with repeated contact attempts (up to 6 per individual) made until sufficient interviews had been completed to reach data saturation.^16^ Timings of interviews were based on participant’s availability, ranging from the final week of pregnancy to 3 months postpartum.

### Data Collection and Analysis

Participants were sent an information sheet then contacted by telephone with interviewees providing verbal consent (audio recorded). Interviews were conducted by an experienced, qualitatively trained female university researcher (MS), lasted approximately 30 minutes, and were audio recorded and transcribed verbatim. Interviewees were usually in their own homes during the interview, often with partners and/or children present in the house. An interview guide with broad, open questions relating to their views and perceived impacts of the MiQuit program was followed with flexibility to deviate according to participants’ interests and opinions.

Data were analyzed thematically^15^; preliminary analysis of initial transcripts identified emerging concepts and enabled refinement of the guiding questions to gain further insight in subsequent interviews. Interviews continued until theoretical saturation was reached with no new concepts emerging.^16^ The stages of analysis involved: immersion in transcripts, developing a coding scheme and coding the data, identification and refinement of themes, amalgamating the extracts from individual transcripts with other examples on the same theme, and further refinement and analysis of themes.^17^ NVIVO 10 software was used to assist with coding and data management with MS and SH double coding all transcripts and FN independently evaluating a third of transcripts to ensure consistency. Particular attention was paid to deviant cases to strengthen validity of the findings.^18^

## Results

Of 203 participants in the trial intervention group, 112 were followed up at the end of pregnancy and 79 (70.5%) gave permission to be contacted for interview. Fifteen participants were interviewed with an age range of 17–37 years and a mean age of 26. At final follow-up, five participants reported abstinence from smoking, with an additional two reporting quitting postpartum. All other participants reported cutting down (Table 1).

**Table 1.** Participant Characteristics

Characteristic	Number
Age band (y)	
17–21	5
22–26	5
27–31	2
≥32	3
Ethnic group	
White British	14
Other Asian—Non-Chinese	1
Number of previous pregnancies	
0	7
1	5
2	0
≥3	3
Relationship status	
Single	3
Partner (smoker)	9
Partner (nonsmoker)	3
Educational level reached	
None	1
GCSE	9
A Level	4
Degree	1
Smoking rate pre-pregnancy (cigarettes per day)	
1–10	2
11–15	3
16–20	6
≥21	4
Smoking rate at interview	
Quit before 38 weeks pregnant (biochemically verified)	5
Quit postpartum (self-report)	2
1–5	3
6–10	4
≥11	1

GCSE = General Certificate of Secondary Education.

Three themes emerged: the perceived “impact” of the text message intervention, how the text message delivery “approach” compares to face-to-face support and views on “optimization”.

## Impact

The daily contact and encouragement provided by texts was considered by most participants to increase their motivation and confidence to quit. Almost all participants reported that the program had an immediate impact by helping break their daily smoking routines and habits, by initially physically distracting them and then causing a delay in smoking by the emotional impact of certain texts.

### Distraction

Checking their phone and having a cigarette was a regular part of the daily routine for almost all the participants. Many identified that the texts helped to keep their hands busy and their mind occupied.

‘I think it was getting stuff on my phone; keeping me busy on my phone…so that I wasn’t distracted somewhere else on a fag…because I’m just one of those where it’s like I’m bored, I’ll have a fag’ Pt 3 (age 25, quit postnatally)

### Emotional Impact

All of the women reported being largely unconcerned about their smoking prior to pregnancy, but expressed feelings of guilt from smoking in pregnancy and a desire to stop/reduce their smoking. As the participants were motivated to quit for their baby, texts about the baby were identified as keeping them firmly focused on their reasons for this quit attempt:

‘Normally I you know, I get 5 minutes, I grab my phone and… go for a fag and then if you read that before you go for a fag, you think oh don’t go for a fag’ Pt 8 (age 30, smoker)

Texts about the stages of development of their baby were widely felt to be interesting, enjoyable and helpful in maintaining their focus:

‘You know, like, ‘Your baby is now the size of a banana’, …They just make you think of the baby and then you’re like, ‘Oh, I don’t need that one. Give the baby a break’ Pt 13 (age 30, smoker)

Texts about the health of the baby and facts about smoking in relation to the baby were reported to engender feelings of guilt in many women. However, the majority of these women felt that this was necessary in improving willpower:

‘There were some texts, I mean they were very disturbing but they were things you needed to know about, what it does to a baby…like obviously smoking that’s going in through your bloodstream – carbon monoxide isn’t it? …they made an impact. I wouldn’t say stop doing them because at the end of the day people need to realise and know what they’re doing’ Pt 2 (age 34, smoker)

However, a minority felt that the feelings of guilt engendered by certain risk texts were counter-productive to their quit attempt and reduced engagement with the program.

‘some of them like made me feel really guilty about it, because I already put a lot of pressure on myself… there was a couple of information texts about what the smoking would do. Like the causes of miscarriage and stuff like that so [that made me feel] guilt and just generally depressed… made me feel so bad that it was just like ‘I don’t want to [get texts] anymore’’ Pt 15 (age 19, smoker)

### Empowerment

Several interviewees suggested that MiQuit helped empower them into making an active choice to quit or reduce their smoking by giving them additional knowledge and support. The combination of the immediate delay/distraction, the emotional impact and the longer term encouragement, appeared to have a cumulative effect in some women that was recognized as improving their motivation and willpower:

‘I think it’s a good idea. It definitely made me think. I didn’t feel so pressurised so it was I was making the choice rather than I was trying to satisfy other people’ Pt 6 (age 34, quit postnatally)

## Delivery Approach

### Constant Encouragement

Every participant reported awareness of societal disapproval regarding smoking in pregnancy, some having experienced negative comments and feeling judged for smoking when pregnant. Positive feedback and encouragement seemed to be lacking in many of the women’s lives, especially in relation to quitting smoking. Many stated that the “text messages” recognized how hard it was for them and were a gentle and non-judgmental yet constant presence. They reported this raised their confidence and self-esteem, and gave them the feeling that “someone is there for them.”

‘You get good feedback and it weren’t just have you quit and you left it at that and just sent us one thing. You kept on sending them and it was positive, so yeah it kept me on the ball…so they keep on you, like they won’t give up with you’ Pt 3 (age 25, quit postnatally)

Further engagement was engendered by the tone and content of the texts being perceived as friendly and encouraging; “a gentle push” rather than forceful.

‘The text messages understand that it’s hard for you to quit smoking…they treat you like you’re mature, like my nan would say to me like how they didn’t come across as like they were like angry …but they did it nicely’ Pt 1 (age 19, smoker)

While the majority felt the tone was suitable, a minority felt it was either too formal or computerized:

‘It felt like I was speaking to a doctor…it’s just I think it should have been something that was a bit, like I say, a bit more personal…from someone I knew, like my mum or anything’ Pt 15 (age 19, smoker)

### Comparison With Face-to-Face Support

Cessation support by text message was felt to be highly convenient and flexible, especially amongst those with three or more children, with many women also stating that it was on “my terms” rather than fitting in with other people’s requirements:

‘The other quitting services, they ring you which is just not convenient. A text message is quickly read and can go back on… The other ones they ring you, give you advice, and then by the time you’ve put the phone down it’s out of your head’ Pt 8 (age 30, smoker)

The regularity and frequency of texts was also felt to be particularly supportive with several stating that receiving support every day was important:

‘I went to the no-smoking clinic at the GP; I’d see her once a fortnight. Well, to me, that’s not supportive. I needed a bit more… it was like somebody was there, sending me a constant, gentle reminder every day: ‘That’s why you’re doing it. This is why you’re quitting’’ Pt 11 (age 37, quit)

The comparison with face-to-face support was not just in connection with the greater convenience of texting, but also in the perception of the difference in the level of pressure exerted on them to quit. There was a widely held view that face-to-face advice would be far more pressurized and adversarial with several women reporting that their experiences of smoking cessation appointments when pregnant were autocratic and made them feel judged and uncomfortable:

‘[The texts] made me feel proud of myself. Like, saying, ‘Oh, the health of the baby’s a lot better’… and stuff like that… [The midwife] I wanted to punch her. It’s hard enough, trying to quit smoking, then to have you yelling at me…The texts don’t pressurise you. They don’t sit there and stare at you thinking, ‘Have you quit yet?’ and you’re, like, being put on the spot’ Pt 10 (age 24, quit)

‘It was nice because it wasn’t too in your face, you didn’t have to communicate with someone… it’s just encouraging you to rather than telling you to… it’s more helpful than someone saying you’ve got to do it’ Ppt 4 (age 23, quit)

In general, the older participants, especially those with more children, were focused on the greater convenience of texts whereas the younger participants (<25 years) were more likely to favor the less judgmental nature of texts over face-to-face support.

Both methods of obtaining cessation support were felt to increase feelings of guilt and a minority expressed the opinion that may be counter-productive. Women who were averse to face-to-face support and being “lectured” reported feeling more in control with texts. However, a minority of participants felt that having greater control could result in them ignoring uncomfortable texts.

‘Face-to-face people make you feel bad because you’re pregnant and you’re smoking… and you’re like well ‘it’s none of your business really… Shut up.’ [Laughing] Yeah, and so if you felt like that with a text, you could just turn it off and put the phone away’ Pt 14 (age 24, smoker)

## Optimization

### Length of Program

Approximately half of participants felt that the texts stopped too early with several stating that they would have liked for them to continue after delivery. Some also considered that the texts stopped too suddenly with insufficient warning leaving them feeling “bereft.” Some of those interviewed postpartum detailed that it was even harder to remain quit after birth due to stress of having a newborn baby and the primary reason for not smoking, their pregnancy, no longer being applicable.

‘The last three weeks for me has been telling because I’ve really wanted a fag ‘cause obviously, like, the birth wasn’t great and everything and I’ve been stuck in the house...I think if I’d had fags in the house I probably would have had one’ Pt 11(age 37, quit)

### Focus on Abrupt Quitting

Several participants did not want to commit to immediately quitting or setting a quit date, perceiving the stress of a quit date might lead them to smoking more. Instead, for some, cutting down to quit was preferred as a quitting strategy; four participants reported achieving abstinence using this method with most of the others reporting substantial reduction in their smoking.

‘If it was more supportive towards cutting down, then- but it was more sort of ‘what’s your quit date? ‘How much have you smoked’…whereas if it was sort of cutting down, it’d be like, you know, ‘try and do this’…it should be more aimed for cutting down I think, to start with’ Pt 5 (age 21, smoker)

### Tailoring

There were preferences for more tailoring, especially in terms of types of texts received and the timings. Many reported “needing” a text as they woke up, with the ideal time specified varying between 3 AM to 9 AM, and felt that not receiving one then jeopardized their quitting chance. The option to tailor the content, especially in relation to the health of the baby, was also suggested

‘well it would be a lot of work but it’s a case of catering to the individual…if people need more help than others or encouraging words rather than guilt words’ Pt 15 (age 19, smoker)

### Enrolment

The vast majority of participants stated that the method used for enrolment into this trial, where a healthcare worker signed them up to receive the text messages, would be far more likely to ensure sign up than an online method or being given a leaflet with the option to sign up following the appointment. This was predominately because of the level of effort required, but some participants also identified that the interest and information from a health professional enhanced their likelihood of engaging:

‘If you’ve got a direct person who’s there and that actually gives you, like, it’s a nice feeling that they’ve approached you… whereas if you just get a leaflet, it’s something that you won’t even probably read, and you’ll pick it up thinking with all good intentions to read it but nine times out of ten you’ll leave it on the side and end up putting it in the recycle bin’ Pt 6 (age 34, quit postnatally)

## Discussion

This study provides the first in-depth view of pregnant women’s perceptions of an SMS text message cessation support program. Participants reported that receiving, reading, and re-reading the texts acted as a physical distraction from smoking and that the content of the texts, particularly messages about the development and health of the baby, were perceived to delay and reduce smoking. Women also reported a strong preference for texts over face-to-face support due to their convenience, frequency and lower likelihood of being perceived judgmental.

This study has several limitations. Transcripts were not checked by participants for accuracy following interview, but their key opinions were summarized and confirmed during interview. As with all retrospective studies we were reliant on the participants’ recall of an intervention they had received, in some cases several months prior to the interview. However, delaying the qualitative interviews until after final follow-up was essential as the interviewing of participants could inadvertently introduce bias to trial findings. As with all interview studies, we may have gained a disproportionate number of interviews with participants who were positive about the study or intervention as those with negative perceptions may be less likely to engage. However, the main barrier to gaining interviews was that the majority of potential participants (over 60%) did not answer their phones, despite multiple attempts. Of those answering, <10% declined to be interviewed and this was almost always due to the inconvenience of fitting an interview around a newborn rather than an unwillingness to engage. The majority of women had other family members present in the house during the interview, which may have impacted on their responses. As all our participants had chosen to join a trial involving text message support, their views on the relative benefits of text over face-to-face may be more extreme than the general population. This was also a relatively young, predominately Caucasian group. These aspects reduce wider generalizability of any comparative judgments except to say that texts are perceived to be preferable by this group of women. Key strengths of this study are that the rigorous COREQ^15^ procedures were followed, transcripts were double coded and success at quitting was biochemically validated. Having access to the main trial’s records enabled purposive selection of participants to ensure a range of experiences and also confirmed there was minimal social desirability bias at qualitative interview, as follow-up information and questionnaire results positively corresponded with views given at interview.

Our findings concerning the high level of acceptability and convenience of texts and greater engagement from tailoring were in line with the MiQuit pilot qualitative study^12^ and MumsQuit, a study of the views of pregnant smokers of an internet-based support program.^19^ Although the mode of delivery differed for MumsQuit, our results also showed that there were individual differences as to preference for harsher messages containing information on negative health outcomes.^19^ While the desire for greater tailoring to allow different timings and quantities of texts is likely to further improve the program’s acceptability, caution is required in tailoring message content. Although reducing risk and health texts may further enhance acceptability for some, it may also reduce impact as these were the ones that were identified as most motivating even amongst those who found them distressing or guilt-inducing. This study found a high level of acceptability, which is important in gaining and retaining engagement. However, the advantages cited over face-to-face support that make texts a more comfortable and acceptable form of cessation support may also reduce effectiveness as they are potentially easier to ignore.

Our findings are in line with the MiQuit acceptability trial^11^ and Quit4baby, a US study^14^ that reported a decrease in quantity smoked and an increase in reported confidence to quit. As with MiQuit, Quit4baby was viewed positively by the majority of participants who also valued the encouragement and social support provided.^14^

As found in the acceptability trial of MiQuit,^11^ the types of text message reported to be of most help with quitting were those concerning risks to the baby of smoking in pregnancy. This was disproportionate to the quantity of texts sent with risk specific information (a maximum of 11% of the texts) yet these and texts about the baby were those cited by participants as the most memorable and impactful. The challenge is delivering such texts without engendering excessive feelings of guilt that could act as a trigger to smoke,^20–22^ or result in the endorsement of beliefs that challenge the need to quit as a means of resolving cognitive dissonance about continued smoking in pregnancy.^23^ The perceived non-authoritarian and friendly manner of the texts perceived by our participants may have facilitated engagement with risk information messages.

There were several areas identified that the women felt would increase the impact of the program. Many participants felt the texts should continue throughout pregnancy and the postpartum period. The postpartum period has been widely identified as a key point for relapse with studies estimating relapse rates between 40% to 90% within 1 year of delivery.^24–27^ Other participants who had quit who were interviewed after birth reported that it was a challenging time for maintaining their quit. This is in line with participants from other studies who reported that stress, especially of adapting to being a parent, caused a return to smoking.^28,29^ Therefore, continuing the program into the postpartum period could be beneficial.

The MiQuit intervention encourages abrupt cessation rather than a process of cutting down. Although cutting down without an intention to fully quit is not advised in the United Kingdom or United States as it has limited health effects,^30^ adapting the program to give a cutting down to quit option may improve engagement and outcomes. One form of cutting down to quit previously trialed is scheduled gradual reduction with evidence reported of its effectiveness in non-pregnant smokers.^31^ Although only a small study, Pollack reports that women following scheduled gradual reduction alongside supportive texts had higher 7-day quit rates at the end of pregnancy than women who received the supportive texts only (13.4% vs. 7.5%)^32^ though no statistical test was undertaken comparing groups.

Having a health professional suggesting and activating sign-up to the intervention during the consultation was almost unanimously considered the most effective method of initiating text message support, with participants suggesting that they would be unlikely to sign up via other methods, as this would involve greater personal initiative.

## Conclusion

Texts were an acceptable method of receiving cessation support with women expressing a distinct preference for texts to be tailored and pregnancy-specific. Participants were positive about how these motivated them to reduce their smoking, particularly via being distracted and through emotional reactions generated by texts focused on the baby. Participants felt texts had key advantages over face-to-face support, largely due to the convenience, the constant presence, encouragement and low-pressure nature of the texts. The overall perception was of being an active participant in the program and using the information to make their own choice to quit, rather than a passive recipient of information and being instructed to quit as many reported feeling in a face-to-face situation.

## Funding

This article presents independent research funded by the National Institute for Health Research (NIHR) under its Programme Grants for Applied Research (reference RP-PG 0109-10020). The views expressed in this article are those of the authors and not necessarily those of the NHS, NIHR or the Department of Health.

## Declaration of Interests

*None declared.*
